# Production of Autoreactive Heavy Chain-Only Antibodies in Systemic Lupus Erythematosus

**DOI:** 10.3389/fimmu.2020.00632

**Published:** 2020-05-05

**Authors:** Shu Xu, Hong Yang, Yue Zhuo, Yangsheng Yu, Hongyan Liao, Song Li, Yinshi Yue, Kaihong Su, Zhixin Zhang

**Affiliations:** ^1^State Key Laboratory of Biotherapy, Sichuan University, Chengdu, China; ^2^Health Management Center, Institute of Health Management, Sichuan Provincial People's Hospital, University of Electronic Science and Technology of China, Chengdu, China; ^3^Department of Pathology and Microbiology, University of Nebraska Medical Center, Omaha, NE, United States; ^4^Department of Laboratory Medicine, West China Hospital, Sichuan University, Chengdu, China; ^5^Department of Medical Oncology, Cancer Center, Qilu Hospital of Shandong University, Jinan, China; ^6^Education-Microbiology/Immunology, Department of Medical Education, California University of Science and Medicine, San Bernardino, CA, United States

**Keywords:** B cell, plasmablast, heavy chain antibody, polyreactive antibody, autoreactive antibody, somatic hypermutation

## Abstract

Systemic lupus erythematosus (SLE) is characterized by the overproduction of high-affinity autoreactive antibodies. Here, we show that more than 65.8% of 222 recombinant antibodies derived from 8 SLE patients can be secreted as heavy chain-only antibodies (HCAbs) when expressed in HEK-293T cells. The secretion of HCAbs follows the conventional endoplasmic reticulum-Golgi apparatus pathway, despite triggering a weaker unfolded protein response (UPR). Many of the purified SLE HCAbs remain autoreactive and have an even higher affinity for dsDNA, Sm, nucleosome, and cardiolipin than HCAbs from healthy individuals. Extended analyses of the CDR3 region and the heavy chain variable (VH) region of HCAb F3 show that the VH region is responsible for IgH secretion, while the CDR3 region determines its reactivity. Such a high frequency of HCAb secretion cannot fully concur with our current understanding of antibody assembly and secretion. The presence of a large proportion of autoreactive HCAbs in SLE reveals a novel mechanism for the generation of autoreactive antibodies in lupus.

## Introduction

Antibodies (Abs), also known as immunoglobulins, are important effector molecules in humoral immunity. Antibodies are exclusively produced by B lymphocytes. At the early differentiation stages of B cells, Abs are expressed as the membrane-bound forms and serve as the B cell antigen receptors. When the development of B cells reaches the terminal differentiation stage, namely, plasma cells, antibodies, are secreted in soluble forms. Normally, Abs are composed of two identical immunoglobulin heavy chains (IgHs) and two identical immunoglobulin light chains (IgLs) linked by disulfide bonds. It is known that antibodies must form a tetramer containing two IgH and two IgL peptide chains to be successfully secreted (1). However, a special category of antibodies, named heavy chain-only antibodies (HCAbs), are secreted as IgH dimers without IgL. An early description of HCAbs appeared in 1964, when γ heavy chain was detected in the serum of a patient with heavy chain disease (HCD), a malignant B cell disease of lymphoplasma cells characterized by the secretion of a large number of IgHs independent of IgLs (2). In addition, a few IgMs presented as μ chain dimers devoid of kappa chains in some HCD patients (3).

HCAbs were commonly regarded as disease derivatives until 1993, when large quantities of IgH antibodies were found in healthy camel serum (4). All HCAbs in camels lack the first constant (C_H_1) domain (5), while the normal IgHs usually have intact carboxyl-terminal regions. For HCAbs, alterations frequently occur in the C_H_1 and V_H_ domains. There is no precise boundary of the peptide deletion; thus, the length of the IgH monomer of HCAb varies between 1/2 and 3/4 of the normal IgH length in most cases (6).

The assembly and secretion process of antibodies has been well-studied. The classical endoplasmic reticulum-Golgi secretion system is regulated by endoplasmic reticulum quality control (ERQC), which strictly controls the secretion of misfolded proteins (7). Simply, overexpression of IgH genes only in mammalian cells can not produce secretory HCAbs. Further studies revealed that the nascent IgH peptides in the ER are associated with a group of molecular chaperone proteins (8). As the most important chaperone, BiP mainly binds to the IgH peptide through interaction with the C_H_1 domain (9). A large number of HCAbs have entire or partial C_H_1 domain deletions, which directly hamper BiP binding. Thus, IgHs can form an IgL-free dimer that can be exported to the extracellular region. Furthermore, it has been reported that IgG C_H_1 folds correctly only upon interaction with the light chain C_L_ domain, indicating that IgL is indispensable in the process of regular antibody secretion (10). However, in some HCD patients, there are still some IgMs that contain only μ chains with complete C_H_1 domains (11). In this regard, the structure responsible for IgH secretion seems to be not confined to the C_H_1 domain.

Systemic lupus erythematosus (SLE) is characterized by an overproduction of high affinity autoreactive antibodies against dsDNA and nuclear antigens (12). However, the mechanism underlying the generation of high affinity autoreactive antibodies in SLE remains unclear. In some clinical cases, the diagnosis of γ-HCD was preceded by SLE (13). In our previous studies, we obtained more than 300 recombinant antibodies from 8 SLE patients by single-cell RT-PCR (separate study). In this study, we expressed and purified 222 recombinant antibodies from 8 patients, and the characterization of these antibodies revealed that many of them can be secreted as HCAbs. We further analyzed the possible features that determine the secretion of HCAbs.

## Materials and Methods

### Study Subjects

Blood samples were collected after written informed consent was obtained in accordance with the University of Nebraska Medical Center (UNMC) Institutional Review Board (IRB). Peripheral blood samples from SLE patients were obtained from the Clinical Research Center at the UNMC. All study subjects were free from other diseases or infections. The controls were all healthy people who did not have ongoing infections or chronic diseases and were matched with the SLE individuals by age, sex, and race. Single-cell RT-PCR analysis was applied in the case of 4 healthy controls and 8 SLE patients. A summary of the demographics of all study subjects is shown in Tables S1, S2.

### Cell Sorting

Peripheral blood mononuclear cells (PBMCs) were isolated from fresh blood samples (20 mL) by density-gradient centrifugation using Ficoll-Hypaque (GE Healthcare Life Sciences). PBMCs were stained with FITC-conjugated anti-human CD3 Abs, BV421-conjugated anti-human CD19 Abs, BV510-conjugated anti-human CD20 Abs, PE-conjugated anti-human CD27 Abs, and APC-conjugated anti-human CD38 Abs (BD Biosciences). CD3^−^CD19^low^CD20^low^CD27^high^CD38^high^ cells were identified as plasmablasts in this study. Plasmablasts were directly sorted into 96-well PCR plates at a single cell/well in 10 μL of lysis buffer (Promega) by a FACS Aria (BD Biosciences). The sorted plasmablasts were directly subjected to RT-PCR or stored at −80°C for further analysis.

### Reverse Transcription (RT)-PCR

The 96-well PCR plate containing sorted cells was incubated at 65°C for 4 h to reverse the crosslinking. IgH, Igκ, or Igλ genes from plasmablasts were amplified by RT-PCR using the OneStep RT-PCR Kit (QIAGEN). A 50-cycle onestep RT-PCR and a 50-cycle nested PCR were performed as previously described (14). After the third round of PCR, the cloned immunoglobulin gene was added and had unique restriction enzyme digestion sites. Then, the final PCR products were subcloned into their corresponding expression vectors, namely Igγ, Igκ, and Igλ plasmids, following the previously established protocol (14–16). All constructed recombinant antibody plasmids were sequenced and analyzed with NCBI IgBLAST.

### Protein Expression and Analysis of Secretion Efficiency

The focus of our study is to explore the secretion feature of heavy chain antibodies, rather than to evaluate the biological function of single heavy chain expression in B cell development. HEK-293T cells have normal secretory pathway as B cells. Besides, HEK-293 was often used to express antibodies (15, 17, 18). Therefore, HEK-293T cells were selected to express antibodies and study the secretion of heavy chain antibodies in our study. Human embryonic kidney cell HEK-293T cells were grown in Dulbecco's modified Eagle medium (DMEM) containing 10% fetal calf serum. Briefly, HEK-293T cells (5 × 10^5^) were transfected with 4 μg plasmids expressing IgH or IgH + IgL. Cells were grown for 72 h and then collected. Two milliliters of culture medium was collected and centrifuged to remove the cell debris, and the secreted antibody in the culture supernatant was incubated with protein A-sepharose beads (BioVision) for 4 h at 4°C. Cell lysate was prepared in 100 μL buffer RIPA (50 mM Tris-HCl pH 7.4, 150 mM sodium chloride, 2 mM magnesium chloride, 0.5% NP-40) containing 1 mM phenylmethylsulfonyl fluoride (PMSF). Then, the samples were centrifuged at 13,500 rpm for 10 min, and the supernatant from the cell lysate was collected for further immunoblotting.

### SDS-PAGE and Immunoblotting

SDS-PAGE was performed on an 8% gel under reducing [1.5% (v/v)] DTT or non-reducing conditions, and gels were stained with Coomassie blue (Pierce). Immunoblotting was performed according to the standard protocols. A 1:2,000 dilution of horseradish peroxidase (HRP)-conjugated goat anti-human IgG antibodies (Abcam) was used for IgH and IgL protein detection. To detect BiP and GRP170, primary anti-human GRP78 antibodies (Abcam, 1:1,000 dilution), and anti-human GRP170 antibodies (Abcam, 1:1,000 dilution) were used, respectively. Bound antibodies were detected by the enhanced chemiluminescence method (ECL) and recorded by a LAS-4000 Imager (GE Healthcare Life Sciences). The intensity of the detected protein bands was quantified by ImageJ. Protein secretion efficiency was determined by normalizing the level of secreted proteins to the amount of expressed proteins in cell lysate.

### Analysis of Secretion Efficiency

The concentration of antibody depends on the intensity of the detected gray scale protein bands. The antibody secretion efficiency was calculated with the following formula: SE = C_m_/(C_m_ + C_l_), where SE = antibody secretion efficiency, C_m_ = antibody concentration in medium, and C_l_ = antibody concentration in lysate. The heavy chains with high secretion efficiency were tested by immunoblotting for three times.

Data analyses were performed using GraphPad Prism version 5 for Windows.

### HCAb Secretion Inhibition Test

HEK-293T cells (5 × 10^5^) were transfected with 4 μg plasmids expressing different IgH genes. Six hours after transfection, cells were washed with serum-free DMEM. Then, for the control and experimental groups, the transfected cells were cultured in AIM medium (Gibco) and AIM medium (Gibco) plus inhibiter (brefeldin A or monensin), respectively. Inhibiter concentration: brefeldin A (10 μg/mL) and monensin (5 μM). The supernatant and cell lysate were collected 24 h post-transfection. Then, the samples were analyzed by immunoblotting.

### Immunoprecipitation

Cells (6 × 10^6^) were washed three times with 1 mL of cold PBS and resuspended in 1 mL immunoprecipitation buffer containing 0.5 M Tris-HCl, pH 7.4, 1.5 M NaCl, 2.5% deoxycholic acid, 10% NP-40, 10 mM EDTA, 1 mM PMSF. After treatment in a prechilled water bath sonifier (Branson 450), the cell lysate was centrifuged at 13,500 rpm for 10 min at 4°C. The supernatant was then mixed with precleaned protein A-sepharose beads (GE Healthcare) and rotated for 12 h at 4°C. Subsequently, the beads were washed three times with immunoprecipitation buffer and once with immunoprecipitation buffer containing 500 mM NaCl, and the associated proteins were eluted. After the addition of 5× protein loading buffer and a heating step of 10 min in boiling water, the samples were then analyzed by immunoblotting. This test was conducted for three times.

### Total mRNA Preparation and Semi-Quantitative RT-PCR

Total mRNA preparation was performed using TRIzol Reagent (Life Technologies), and cDNA was subsequently prepared using the PrimeScript® RT Reagent Kit (TaKaRa) according to the manufacturer's protocol. One microliter of cDNA was mixed with 13 final concentrations of Fast SYBR Green Master Mix (Applied Biosystems, Life Technologies) and 0.2 pmol/mL final concentrations of the respective forward and reverse primers in a total volume of 10 μL in a MicroAmp optical 96-well-plate (Applied Biosystems). BiP: F, 5′-CATCACGCCGTCCTATGTCG-3′ and R, 5′-CGTCAAAGACCGTGTTCTCG-3′; ATF4: F, 5′-CCCTTCACCTTCTTACAACCTC-3′ and R, 5′-TGCCCAGCTCTAAACTAAAGGA-3′; CHOP: F, 5′-GAACGGCTCAAGCAGGAAATC-3′ and R, 5′-TTCACCATTCGGTCAATCAGAG-3′; Derlin: F, 5′-TACGCGACTTGAAACAGGAGC-3′ and R, 5′-AGCCAGTAATCACGATGCAAA-3′; ERdj3: F, 5′-TTGTCGGCAAGAGATGCGG-3′ and R, 5′-CCAGCGTTCGTTCTTCATTCA-3′; ERdj4: F, 5′-TCTTAGGTGTGCCAAAATCGG-3′ and R, 5′-TGTCAGGGTGGTACTTCATGG-3′; ERdj5: F, 5′-TGCAGCATGTTAGAAGTACAGTG-3′ and R, 5′-AGCCAGCCAATACCAGCAG-3′; ERdj6: F, 5′-GGATGCAGAACTACGGGAACT-3′ and R, 5′-TCTTCAACTTTGACGCAGCTT-3′; and HERP: F, 5′-TGCTGGTTCTAATCGGGGACA-3′ and R, 5′-CCAGGGGAAGAAAGGTTCCG-3′.

The reaction was performed in a CFX96 Real-Time PCR System (Bio-Rad). For quantitative RT-PCR, the reaction was performed in 0.2 mL PCR tubes (Biozym) using HT Master Taq polymerase, 0.2 mM dNTPs, MgCl_2_-containing PCR buffer (Genaxxon Bioscience), and 1.5 mM forward and reverse primers. This test was conducted for three times.

### Antibody Production and Purification

Plasmid DNA (10 μg) expressing IgH only or the IgH + IgL genes was cotransfected into 90% confluent HEK-293T cells using polyethylenimine reagent (PEI, Sigma-Aldrich) in Opti-MEM® Reduced-Serum Medium (Life Technologies). Six hours after transfection, cells were cultured in AIM medium (Gibco) without serum. The supernatant was collected after 3 days of culture. Antibodies were purified with protein A-sepharose beads.

### ELISA

For the polyreactivity ELISA, microplates (Immunoplates, Corning, USA) were coated with 10 μg/mL of double-stranded DNA (dsDNA) (Sigma-Aldrich), Sm (Meridian), nucleosome (Meridian), or cardiolipin (Sigma-Aldrich) in coating buffer (pH = 7.6) at 4°C overnight. Coated plates were blocked with 100 μL PBST/BSA buffer (2.0% BSA, 0.05% Tween-20) for 2 h at RT, followed by incubation with 100 μL of purified antibodies (1:4 serially diluted in PBS with 2% BSA) at RT for 1 h. After washing four times with PBST, horseradish peroxidase (HRP)-labeled goat anti-human IgG (H + L) antibodies (Proteintech, USA) were added 100 μL (1:3,000 dilution) to each well and incubated at RT for 1 h. For color development, 3,3′,5,5′-tetramethylbenzidine (TMB, Sigma-Aldrich) was added and incubated for 10 min. The reaction was stopped with 2 M H_2_SO_4_, and the absorbance at 450 nm (OD_450_) was measured on a Biotek plate reader (Biotek, USA). This test was conducted for three times.

### Antinuclear Antibody (ANA) Test

Immunofluorescent ANA assay (IFA) was performed using HEp-2 cells. Before the ANA test, HEp-2 cells were plated on slides at 2 × 10^4^ cells in a 24-well-plate and cultured overnight. The next day, HEp-2 cells were washed with PBS and then fixed with 3.7% paraformaldehyde for 30 min on ice. HEp-2 cell-coated slides were blocked with PBST buffer (10% goat serum, 0.3% Triton) for 30 min at RT, followed by incubation with purified antibodies (25 μg/mL) for 2 h at RT. After washing with PBS 4 times, slides were incubated with FITC-labeled goat anti-human IgG Abs (Abcam, UK) and DAPI (all 1:100 dilution) for 1 h at RT. Slides were washed with PBS and visualized under a confocal fluorescence microscope (FV1000, Olympus). Images were captured with the same exposure setting and analyzed using a confocal fluorescence microscope (FV1000, Olympus). Specific staining patterns, such as nuclear and cytoplasmic staining, were documented.

## Results

### Secretion of Recombinant HCAbs Derived From SLE Patients

**Figure 1:**
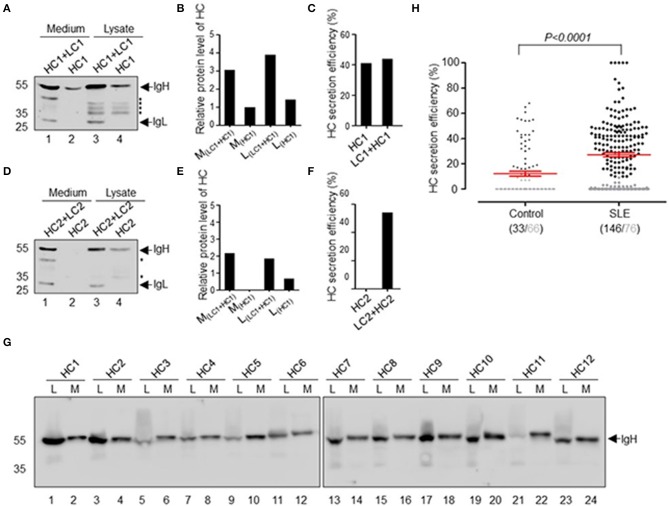
Secretion of recombinant HCAbs derived from SLE patients. **(A–F)** Secretion differences between conventional antibodies and HCAbs. HC1 is the SLE HCAb heavy chain, and HC2 is the conventional heavy chain and these heavy chains are paired with the light chains LC1 and LC2, respectively. Western blot analyses of HC1 **(A)** or HC2 **(D)** in culture medium or cell lysate. The asterisk refers to the broken heavy chain. Their relative expression levels are shown in **(B)** or **(E)**. The HC secretion efficiency is shown in **(C)** or **(F)**. **(G)** Twelve representative HCAbs screened from SLE patient antibodies, L refers to lysate, and M refers to medium. **(H)** Secretion efficiency of 222 IgHs derived from SLE patients and 99 IgHs derived from healthy individuals. The gray points and gray numbers refer to IgHs with secretion efficiency below 10%. The *P*-value was determined by Mann-Whitney *U*-test.

In our previous studies, we obtained approximately 300 recombinant antibodies from SLE patients. During our expression and purification of these recombinant antibodies, we found that some of them can be secreted as HCAbs. For example, HC1 is an SLE-derived IgH encoding HCAb, and HC2 is a conventional IgH, both of which can be paired with IgL for secretion (Figures 1A,D). HEK-293T cells were transfected with plasmids encoding the IgH or IgH + IgL genes. After 72 h, we analyzed the antibody expression levels in culture supernatant and cell lysate with immunoblotting. IgH HC1 can be expressed and secreted with or without IgL LC1 (Figures 1A–C). The secretion efficiency of HCAb HC1 is similar to that of HC1+LC1 (Figure 1C). Such results are different from HC2, which cannot be secreted independently from LC2 (Figures 1D–F).

Further analysis of 222 recombinant IgH genes derived from 8 SLE patients and 99 IgH genes derived from 4 healthy donors revealed that many of them have the capacity for IgL-independent secretion (Figures 1G,H). The IgL-independent secretion efficiency of IgH in SLE patients was significantly higher than that in healthy donors (*p* < 0.0001). In summary, we defined the secretion efficiency of IgH >10% as IgL-independent secretion of HCAb. Approximately 65.8% (146/222) of SLE-derived IgH genes can be expressed and secreted as HCAb independent of IgL. The secretion efficiency of IgH ranges from 10 to 100% (Figure 1H). The proportion of ruptured cells was <0.3% after 72 h of transfection, which means that the HCAbs detected in the culture supernatant were not due to ruptured cells.

### Expression and Secretion of SLE HCAbs

**Figure 2:**
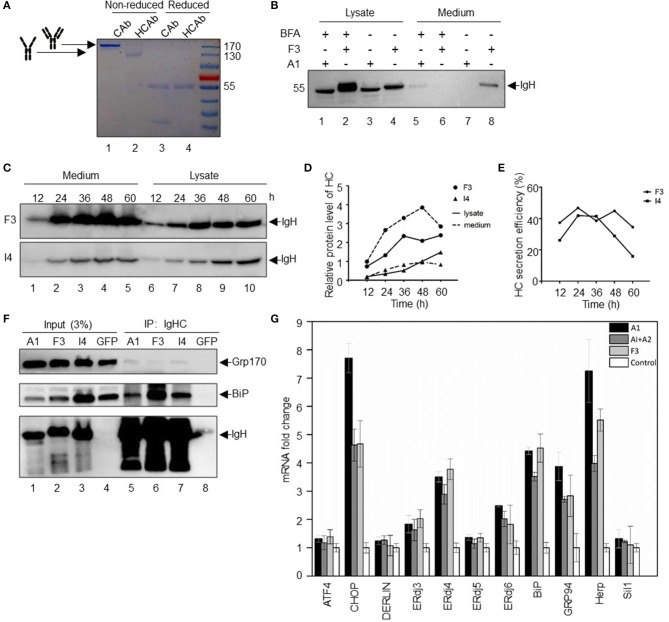
Expression and secretion of SLE HCAbs. **(A)** Secretion forms of HCAbs and conventional antibodies. Reduced with DTT and non-reduced with no DTT, stained with Coomassie brilliant blue after SDS-PAGE. **(B)** SLE HCAbs are assembled and transported through the ER-Golgi protein transport and secretion pathway. BFA is brefeldin A, a vesicle-mediated protein transport inhibitor from ER to Golgi. **(C–E)** Changes in the protein expression and secretion of HCAbs within 60 h. F3 is a HCAb with high protein expression and secretory efficiency, while I4 is relatively weaker in both aspects. **(F)** HCAbs bind to BiP and GRP170 to varying degrees. The binding ability of heavy chain to important chaperone molecules of the endoplasmic reticulum was analyzed by an immunoprecipitation assay. F3 and I4 are HCAbs, and A1 is a conventional IgH. **(G)** Expression of HCAbs, IgH, or conventional antibodies induced different endoplasmic reticulum gene expression. Fluorescence quantitative real-time PCR was used to analyze the expression of important genes involved in protein folding, assembly and degradation in the endoplasmic reticulum after 24 h of transfection of different constructs (mean ± s.e.m., *n* = 3 independent experiments). A1 is a non-secretory IgH, A2 is an IgL, and F3 is an HCAb IgH.

For all IgG molecules, the typical assembly form is an [HC]_2_[LC]_2_ tetramer. As an initial study to analyze the subunit components of HCAb, we performed non-reduced and reduced SDS-PAGE using conventional Abs and HCAbs (Figure 2A). In denatured but non-reducing condition, IgG molecules migrate at 180 kDa (Figure 2A, lane 1), while the HCAbs migrate at 110 kDa (Figure 2A, lane 2). In denatured and reducing condition, IgG molecules can be separated into two bands at 55 and 30 kDa, corresponding to the IgH and IgL polypeptides, respectively (Figure 2A, lane 3), while the HCAbs have only one 55 kDa band (Figure 2A, lane 4), which indicates that the HCAbs only contain IgH polypeptides.

The conventional protein secretion pathway is through the endoplasmic reticulum-Golgi system (19), and there are other unconventional secretion pathways in mammalian cells, such as misfolding-associated protein secretion, which uses the ER-associated deubiquitinase USP19 to preferentially export aberrant cytosolic proteins (20). To evaluate the secretion pathway adopted by the HCAbs, the inhibitor brefeldin A (BFA) was used to block Ab transport from the endoplasmic reticulum to the Golgi apparatus. BFA can inhibit the formation of vesicles. Thus, the secretory protein in the canonical ER-Golgi secretion pathway, which depends on vesicular transport, is retained in the ER and cannot be secreted in further step (21). Two HCs were selected in these studies: F3 with strong secretion ability and A1 with no secretion ability (Figure 2B). Notably, BFA treatment prevented HCAb F3 secretion (Figure 2B, lane 6), and the amount of F3 that accumulated in the cells treated with BFA (Figure 2B, lane 2) was significantly higher than that in the cells not treated with BFA (Figure 2B, lane 4). Although there is a very low level of A1 IgH in the supernatant upon BFA treatment (Figure 2B, lane 5), the A1 IgH cannot be secreted without IgL in the absence of BFA (Figure 2B, lane 7). Further, two other HCAbs (F9 and H6) with high secretion capacity have the same behavior as F3 in BFA secretion inhibition test (Figure S1A). BFA inhibits protein secretion at an earlier step by blocking protein transport from ER to Golgi apparatus. Differently, monensin, another protein secretion inhibitor, can block the protein transport from Golgi apparatus to extracellular environment by collapsing intracellular Na^+^ and H^+^ gradients necessary for protein transport. Then we used monensin to explore whether the Golgi apparatus is the organelle that HCAbs (F9 and H6) have to pass through during secretion (22). The result showed that monensin can block the secretion of F9 and H6 (Figure S1B). In summary, the secretion of HCAbs are sensitive to BFA and monensin treatment, indicating that the secretion of HCAbs is still through the traditional endoplasmic reticulum-Golgi apparatus pathway.

Antibody expression and secretion is a continuous process. Next, we analyzed the expression of HCAbs in the cytoplasm and supernatant at different time points. Two HCAbs were selected: F3 with a strong secretion ability and I4 with an intermediate secretion ability. Within 60 h, the expression of IgH in the cytoplasm and supernatant changed continuously (Figure 2C). The secretion of HCAb was sustained for 48 h, but the amount of Ab in the supernatant decreased at 60 h (Figure 2D), which may be due to nutrient exhaustion and protein degradation in the supernatant. The secretion efficiency of HCAbs also decreased after 24 h (Figure 2E).

Deletion of the C_H_1 domain may result in light chain (LC)-independent HCAb secretion, similar to camel HCAbs. Without the C_H_1 domain, the IgH dimers fail to bind to BiP and can be secreted freely (10). To determine whether the SLE-derived HCAbs are still able to bind to BiP, we performed immunoprecipitation assays with the three HCs described above: A1, F3, and I4. The immunoprecipitation results showed that all three peptides can bind to BiP (Figure 2F). These three peptides also bind weakly to GRP170, the cochaperone of BiP. This weak binding force indicates that HCAbs may interact with GRP170 in an indirect way. GRP170 is a nucleotide exchange factor (NEF) that delivers ATP to BiP and promotes BiP dissociation from the substrate to complete the binding-release-binding cycle of BiP, thus facilitating the folding and maturation of nascent protein (23). The interaction with BiP and GRP170 suggests that SLE HCAbs are involved in the routine ER protein folding and maturation process and the IgL-independent secretion of HCAbs may be due to other unknown mechanism in addition to the C_H_1 domain deletion mechanism.

The accumulation of misfolded proteins in the ER will induce the unfolded protein response (UPR) (24) and activate several associated ER genes (25). To further investigate the mechanism responsible for the IgL-independent secretion of SLE-derived HCAbs, we evaluated the expression of the ER stress gene upon overexpression of A1, A1 + A2 (IgH + IgL), and F3 in HEK-293T cells. Overexpression of A1 strongly induced *CHOP, ERdj4, BiP, GRP94*, and *HERP* expression (Figure 2G). Overexpression of A1 + A2 led to a reduced induction of *CHOP, GRP94*, and *HERP* expression. Compared to cells overexpressing A1 + A2, cells overexpressing F3 showed similar levels of induction of *CHOP, GRP94*, and *HERP*. These results indicate that overexpression of HCAbs did not trigger a strong UPR.

### Many HCAbs From SLE Patients Are Autoreactive

**Figure 3:**
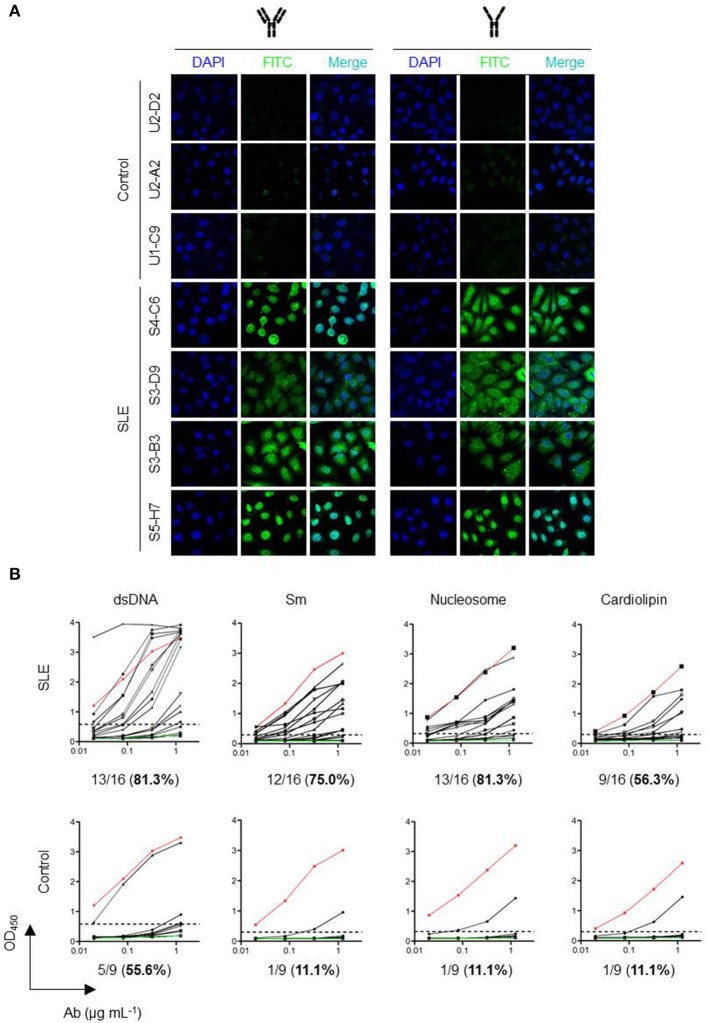
Many HCAbs from SLE patients are autoreactive. **(A)** Autoreactivity (ANA) of HCAb. HEp-2 cells were used as immunofluorescence targets. The nuclei were stained with DAPI. FITC-conjugated goat anti-human IgG was used as a secondary antibody. **(B)** Polyreactivity of HCAbs ELISA results of 25 HCAbs against double-stranded DNA (dsDNA), Smith antigen (Sm), nucleosome, and cardiolipin. The antibodies were used at 0.02, 0.078, 0.313, and 1.25 μg/mL. Red and green lines indicate positive and negative controls, respectively. Dashed lines represent the cut-off OD_450_ value for positive reactivity. Percentage in brackets refers to the positive rate.

Systemic lupus erythematosus is characterized by the production of high affinity autoantibodies in the serum (26). We performed immunofluorescence microscopy studies using fixed HEp-2 cells to determine whether the purified HCAbs are autoreactive. The selected 4 antibodies derived from SLE patients were expressed and secreted as complete [HC]_2_[LC]_2_ tetramers and strongly reacted with nuclear antigens on HEp-2 cells; the 4 purified HCAbs expressed and secreted as [HC]_2_ also strongly reacted with nuclear antigens. HCAbs from healthy individuals displayed almost no reactivity against nuclear antigens in both formats (Figure 3A).

We selected 16 HCAbs from SLE patients and 9 HCAbs from healthy individuals and analyzed their polyreactivities against SLE autoantigens (dsDNA, Sm, nucleosome, and cardiolipin) (Figure 3B). HCAbs from healthy individual and SLE patients all have high anti-dsDNA positive rates (55.6 and 81.3%). However, when referring to the other three antigens, the SLE HCAbs showed obviously higher positive rate than healthy individual HCAbs, especially to Sm (75.0%) and nucleosome (81.3%). These results indicated that all these HCAbs have immunological functions. Moreover, the contribution of SLE HCAbs to autoreactivity could not be ignored.

### Sequence Analysis of SLE HCAbs

**Figure 4:**
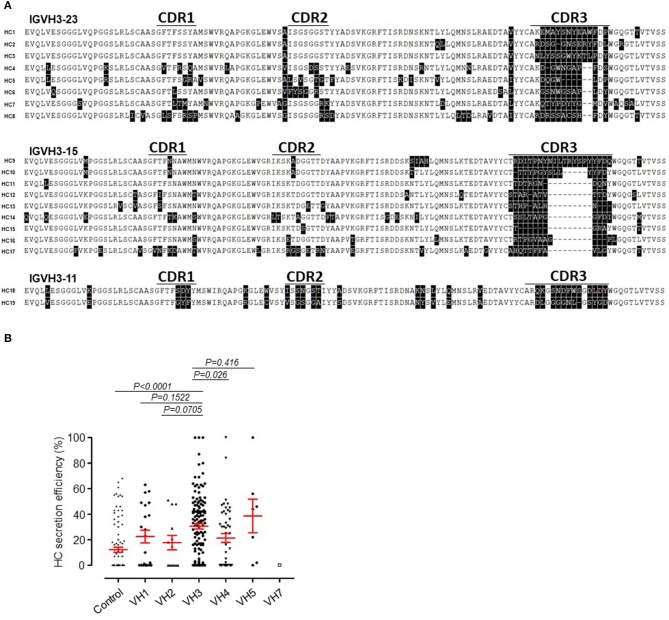
Sequence inconsistency and usage of VH family genes in SLE HCAbs. **(A)** No obvious sequence similarity among 19 HCAbs encoded by three VH germline genes in CDR3. **(B)** Comparison of the secretion efficiency of SLE HCAbs encoded by 6 VH germline gene families. The *P*-value was determined by Mann-Whitney *U*-test.

Sequence alignment of the protein primary structure was carried out for 19 SLE HCAbs, which were divided into three groups according to the germline genes (Figure 4A). We found that all the shared sequences were located within the structural components of these antibodies, including regions encoded by the VH and JH genes. The hypervariable region CDR3 of these HCs showed almost no homology among these HCAbs, and the length of CDR3 also showed no obvious consistency.

Based on their VH germline gene usage, we divided 222 HCAbs derived from SLE into the following groups: VH1, VH2, VH3, VH4, VH5, and VH7. The secretion efficiency of the IgH-containing VH3 gene was significantly higher than that of the VH4 gene (Figure 4B) but was not significantly different from those of the other VH genes due to the lower number of IgH-containing VH family genes.

In addition, all SLE heavy chains were divided into secreted and non-secreted heavy chain group according to the cutoff value (10%) specified in Figure 1H. We analyzed the usage of VH, DH, and JH gene between the two groups. For VH genes, the VH3-7, VH3-15, VH3-21, VH3-23, and VH4-39 genes were used more frequently in HCAbs (Figure S2A). For DH genes, the DH1-7, DH2-2, DH2-21, and DH7-27 genes were used more frequently in HCAbs (Figure S2B). The JH genes showed no preference in SLE HCAbs (Figure S2C). For all SLE HCAbs, the length of CDR3 varies from 5 to 27 AA (Figure S2D). The average secretion efficiency of the group with the shortest CDR3 (<11 AA) is higher than other three groups (Figure S2D). However, no significant difference exists between any two of the four groups (*p* > 0.05).

### CDR3 Alterations in HCAb Affect Its Autoreactivities but Not Its Secretion

**Figure 5:**
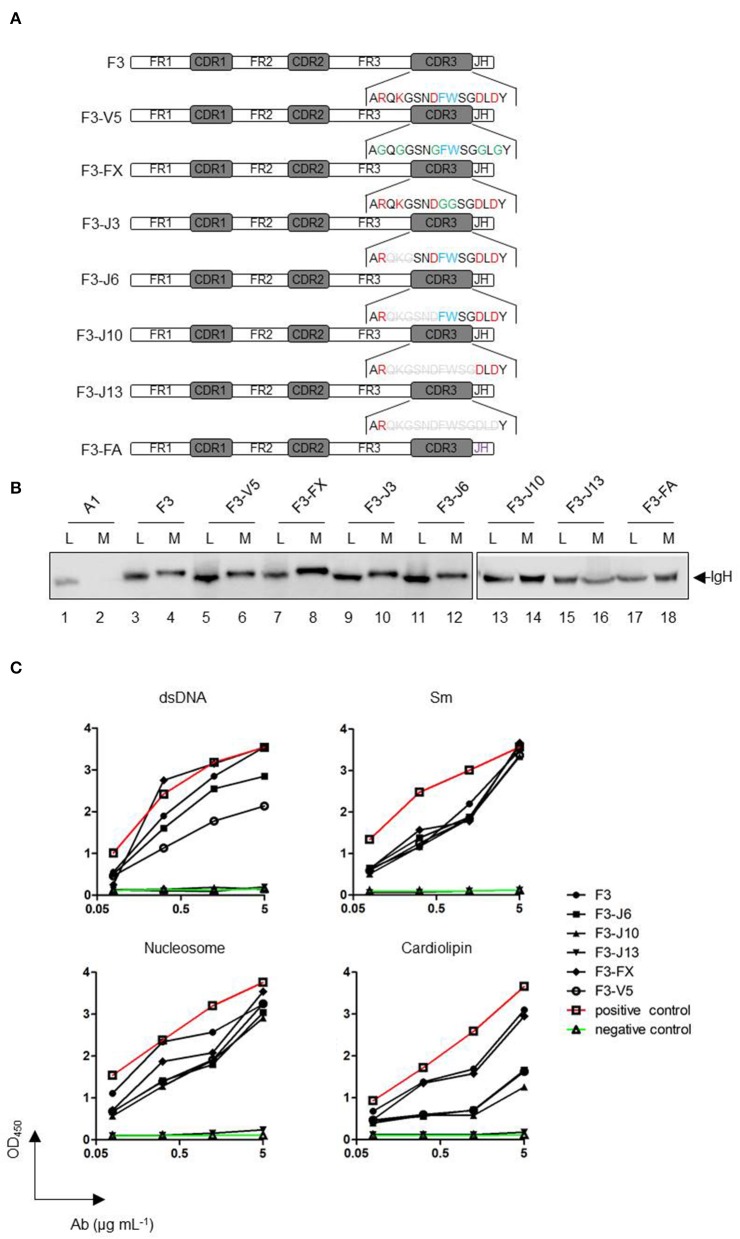
Alterations of CDR3 in HCAbs affect their autoreactivities but not their secretion. **(A)** A schematic diagram of the original and CDR3 mutant antibodies. A1 is a conventional IgH, and F3 is a HCAb IgH. The red font amino acids of CDR3 are charged amino acids, the blue font amino acids represent amino acids containing side chains of aromatic groups, and the green font amino acids represent mutated amino acids. Gray amino acids with deletion lines represent the amino acids deleted in the mutation. All mutations were derived from HCAb F3. F3-V5, mutation of six charged amino acids into glycine; F3-FX, mutation of two aromatic amino acids into glycines; F3-J3, starting from the first amino acid after arginine, three amino acids are deleted from the amino terminus to the carboxyl terminus; F3-J6, F3-J10, and F3-J13 are deleted accordingly in accordance with the above deletion manner. F3-FA replaces the JH sequence of F3 with that of A1 (purple). **(B)** Secretion efficiencies of F3 CDR3 mutant heavy chains. **(C)** Changes in the polyreactivity of HCAb F3 CDR3 mutants. dsDNA, Smith antigen (Sm), nucleosome, and cardiolipin were used as antigens. The antibodies were used at 0.078, 0.313, 1.25, and 5 μg/mL.

To determine the region that is responsible for the secretion of HCAbs, we carried out point mutations and serial deletion mutations in the CDR3 region of HCAb F3 (shown in the schematic diagram in Figure 5A). All mutations showed only a subtle effect on the rate of HCAb F3 secretion, indicating that CDR3 did not influence the secretion capacity of HCAb F3 (Figure 5B). It should be noted that all of the deletion mutants kept the alanine (A), arginine (R), and tyrosine (Y) residues intact, which determined the stability of the antibody structure. Thus, these mutants would not be recognized as misfolded proteins by the ERQC system, and no significant change was observed in HCAb mutant expression.

The IgH CDR3 regions are crucial for antigen recognition. Previous studies have shown that autoreactive antibodies have two features: long IgH CDR3 and positively-charged amino acids (14). In the case of autoreactive HCAb F3, either mutation of the charged and aromatic amino acids in CDR3 or the shortening of CDR3 affects its autoreactivity and polyreactivity (Figure 5C). The affinity changes in these mutants to the antigen appeared to be proportional to the degree of mutation. For Sm and nucleosome, the absence of charged amino acids and aromatic amino acids in CDR3 did not change the binding ability of F3. Even when about 2/3 of CDR3 was deleted, the mutant (F3-J10) still maintained the equal level of reactivity as F3. Eventually, the complete loss of reactivities in F3-J13 indicated that the antigen binding sequence present in the C-terminal of CDR3. Differently, the amino acids, which determine anti-dsDNA and anti-cardiolipin reactivities, are widely distributed in F3 CDR3. The results showed that the loss of charged amino acid could obviously alter the reactivities against dsDNA and cardiolipin. Moreover, as the length of CDR3 became shorter, both of anti-dsDNA and anti-cardiolipin reactivities gradually decreased. In conclusion, CDR3 alteration did not affect the secretion of HCAb F3 but reduced its polyreactivity.

### Changes in the VH Gene Affect the Secretion of HCAb

**Figure 6:**
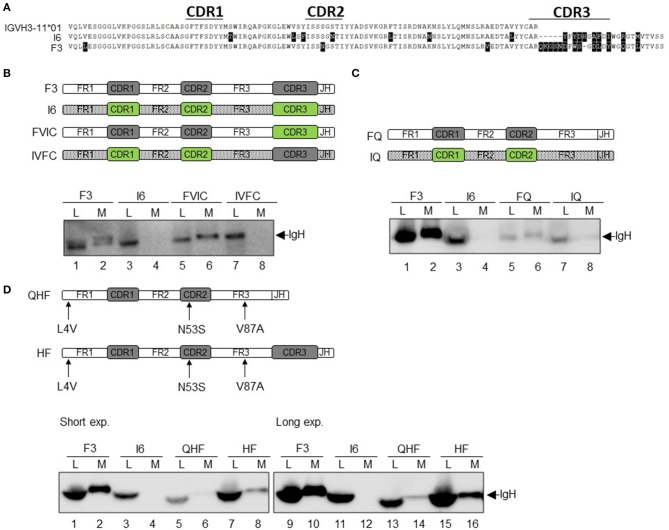
Changes in the VH gene affect the secretion of HCAb. **(A)** Sequence alignment of heavy chain F3 and I6. F3 and I6 are encoded by the same VH germline gene (different from the position of somatic mutations). **(B)** Correlation of the VH region with the secretion of HCAb. The CDR3 regions of F3 and I6 were completely interchanged and recombined into FVIC and IVFC, respectively. **(C)** CDR3 determines the level of IgH protein expression but does not affect the secretory capacity. CDR3s of F3 and I6 were completely removed and recombined into FQ and IQ, respectively. **(D)** Somatic mutations in the VH gene alter the secretion of heavy chain antibodies. Three mutant amino acids produced by somatic mutations in the VH gene coding region of F3 were mutated back to amino acids in the germline gene and recombined into HF. CDR3 of HF was completely excised, and HF was recombined into QHF. *Short exp*, normal exposure condition; *Long exp*, long exposure condition.

To investigate whether the VH gene-encoded region is responsible for the IgL-independent secretion of HCAb, we selected two SLE antibodies encoded by the same VH gene VH3-11^*^01: F3 with strong LC-independent secretion capacity and I6 with no LC-independent secretion capacity (Figure 6A). Compared to the germline VH3-11^*^01 sequence, F3 and I6 have 3 and 7 different point mutations, respectively. The CDR3 lengths of F3 and I6 are 16 amino acids (AA) and 12 AA, respectively.

First, the CDR3 regions of I6 and F3 were interchanged to generate two recombinant antibodies, FVIC and IVFC, respectively (Figure 6B). FVIC combines the VH and JH coding sequences of F3 with the CDR3 sequence of I6, while IVFC combines the VH and JH coding sequences of I6 with the CDR3 sequence of F3. The results showed that FVIC could be secreted without IgL in a manner similar to F3 secretion, while IVFC could not (Figure 6B, lanes 6 and 8).

Then, we constructed the two truncated mutants FQ and IQ by completely deleting the CDR3 of F3 and I6, respectively. The expression of these two HCs in the cytoplasm decreased compared to the original expression of F3 and I6; this result may be due to the protein structure changes after the removal of CDR3, including alanine (A), arginine (R) and tyrosine (Y) (Figure 6C, lanes 5 and 7). Although the expression of the FQ protein is severely reduced, FQ could still be secreted in a certain proportion (Figure 6C, lane 6). These results suggested that IgL-independent secretion of HCAb is determined by the VH region.

Compared to the germline sequence of VH3-11^*^01, F3 has three-point mutations: L4, N53, and V87 in FR1, CDR2, and FR3, respectively (Figure 6D). We reversely mutated these three residues in F3 to the corresponding residues in the germline gene sequence (L4V, N53S, and V87A), and the new construct was called HF. Moreover, to exclude the interference of CDR3 on secretion, the CDR3 of HF was completely deleted, and the construct was named QHF. As shown in Figure 6D, lane 8, the secretion efficiency of HF was greatly reduced compared to that of F3, but was not completely blocked (Figure 6D), indicating that the high efficiency of IgL-independent secretion was somehow determined by these 3 amino acids in the VH gene. Deletion of CDR3 caused a significant decrease in cytoplasmic QHF protein expression (Figure 6D, lane 5). However, at longer exposure time, we can still see a certain degree of QHF secretion (Figure 6D, lane 14). Even at longer exposure time, there was still no detectable I6 protein in the supernatant (Figure 6D, lane 12). Comparing the relative levels of HF (lanes 7–8) to QHF (lanes 13–14), we found the secretion efficiency was nearly identical, which confirmed that the CDR3 had no effect on the secretion of HCAb F3 (Figure 5B). Taken together, these results indicate that the three mutations within the VH3-11 gene of F3 are responsible for the efficient secretion of HCAb.

## Discussion

The conventional antibody contains two IgH peptides and two IgL peptides. The expression, assembly, and secretion of antibodies are tightly controlled (9). However, in the analyses of 222 IgH genes derived from SLE and 99 IgH genes derived from healthy individuals, we found that many IgH genes derived from healthy individuals and SLE patients can be expressed and secreted independently of IgL when transiently expressed in HEK-293T cells. HCAbs are widely found in recombinant antibodies derived from healthy individuals and SLE patients. In particular, 65.8% of IgH derived from SLE patients can be secreted independently of IgL with varying secretion efficiency. The SLE HCAb is composed of two full-length IgH peptides with a molecular weight of 110 kDa. Our finding can not fully follow the current knowledge that the assembly of IgH with IgL is the prerequisite for antibody secretion (27). Previous studies have shown that IgLs also have more than one type of secretion form. Despite the fact that IgLs can be secreted freely (28), there are still some IgLs whose secretion depends on an association with IgH (29). In humans, the IgL-independent secretion of IgH may be a previously neglected phenomenon.

Many kinds of HCAbs have been reported (30–33). The proportion of HCAbs varies in Camelidae. In camels, HCAbs account for 50–80% of serum antibodies, indicating that HCAbs play an important role in the immune defense systems of these animals (34). The common feature of these Camelidae HCAbs is that they are devoid of C_H_1. On the basis of IgH, there are 3 types of HCD, including α-HCD, γ-HCD, and μ-HCD. Similar to Camelidae, HCD patients have HCAbs that lack C_H_1, and most of those HCAbs have obvious deficiencies in variable domains in addition to the C_H_1 deletions (35). Although HCAbs have great diversity, the major characteristic is the C_H_1 deficiency, and C_H_1 contains cysteine to form covalent bonds with IgLs. Therefore, it was concluded that the absence of C_H_1 is the main factor responsible for the IgL independent secretion of HCAbs. The interaction of BiP with unstructured C_H_1 was proven to be a decisive step in HCAb secretion (10). Even so, the HCAbs of some HCD patients lack only the V_H_ domain (3, 11). In our studies, HCAbs derived from the peripheral blood plasmablasts of SLE patients have the complete domain organization of IgH from conventional IgG1, including the C_H_1 domain, because the constant region was encoded by the same expression vector for all these IgH genes. The expressed IgH peptides are still able to bind to BiP. The assembly and secretion of the SLE HCAbs trigger a substantially decreased level of UPR than the assembly and secretion of conventional IgHs, indicating that the structural conformations of HCAbs are not recognized as misfolded proteins by ERQC. These studies show that these SLE HCAbs have the general characteristics of traditional tetramer antibodies.

Due to the lack of IgL, HCAbs may have altered biological functions. In our study, a large portion of the purified SLE HCAbs are autoreactive against nuclear antigens or a selected panel of autoantigens, indicating that these secretory HCAbs are fully functional as the pathogenic SLE autoantibody with conventional form. These findings suggest that the production of HCAbs in SLE may be a novel mechanism to generate high affinity pathogenic autoantibodies. We speculate that at the early stage of B cell development, IgL-independent expression of IgH may bypass the negative selection provided by IgL gene editing; such B cells may produce autoreactive antibodies later upon activation. In germinal center reactions, somatic mutation may disrupt functional IgL genes, and B cells expressing IgH only may survive.

We have adopted a series of mutation approaches to reveal the factors affecting the secretion of HCAbs. Analyses of the CDR3 mutants of F3 showed that although the CDR3 region was important for autoreactivity, it was not responsible for IgL-independent secretion. This notion was further confirmed by the interchanging of CDR3 between F3 and I6. Both IgHs contain VH3-11 genes, but F3 is a HCAb, while I6 is not. Interestingly, the F3 VH region is responsible for IgL-independent secretion, while the I6 VH region is not, most likely because I6 carries different mutations. When the F3 sequence was reversely mutated back to the germline sequence, the secretion efficiency was dramatically reduced. We also noticed that the VH3-11 germline sequence-encoded IgH still has weak self-secretion capacity, suggesting that certain VH germline genes have the potential to be expressed independent of IgL and produce HCAbs. Somatic hypermutation may further enhance or eliminate their self-secretion efficiency. For example, it is reported that the wild type single-stranded T-cell receptor scTCR could not be secreted in yeast, but after point mutation, soluble TCR appeared in the supernatant of culture medium, and its thermal stability was higher than that of non-secreted scTCR (36).

Considering the evolution of antibody, the conventional structure with two IgH and two IgL peptides might be the mainstream, which provides effective function and stable structure. However, different functional requirements may select different structural antibody products. For example, to help camel survive in the hot desert environment, their antibodies are evolved into small molecular antibodies lacking C_H_1 domains, and the proportion of HCAbs counts up to 80% (34). HCAbs may have a more stable structure and may be more useful for binding special antigens. In this regard, our finding shows that a large portion of SLE-derived IgH genes can produce HCAbs, and many of these HCAbs are both autoreactive and polyreactive. We speculate that, during the chronic inflammatory response in SLE patients, the IgH VH genes may be heavily mutated, thus generating many HCAbs. This provides an explanation for the significant difference in the production frequency of HCAbs between SLE patients and healthy people. The production and biological significance of such HCAbs in SLE is worthy of further investigation. In addition, hence mutation may produce HCAbs, we also speculate that a high frequency of HCAbs may be generated during other immune responses, such as antibacterial or antiviral responses. Such HCAbs may have important functions in recognizing special viral or bacterial antigens, which contribute to humoral immune defense.

## Data Availability Statement

The raw data supporting the conclusions of this article will be made available by the authors, without undue reservation, to any qualified researcher.

## Ethics Statement

Blood samples were collected after written informed consent was obtained in accordance with the University of Nebraska Medical Center (UNMC) Institutional Review Board (IRB).

## Author Contributions

ZZ and KS designed the experiments. SX and HY conducted the experiments. YYu, HL, SL, and YYue provided expression constructs. YZ provided useful guidance and discussions for the experiment. All authors worked together wrote the manuscript.

## Conflict of Interest

The authors declare that the research was conducted in the absence of any commercial or financial relationships that could be construed as a potential conflict of interest.

## References

[1] Mains PE Sibley CH. The requirement of light chain for the surface deposition of the heavy chain of immunoglobulin M. J Biol Chem. (1983) 258:5027–33. 6403541

[2] Franklin EC Lowenstein J Bigelow B Meltzer M. Heavy chain disease—a new disorder of serum γ-globulins: report of the first case. Am J Med. (1964) 37:332–50 10.1016/0002-9343(64)90191-3 14209281

[3] Franklin EC Frangione B Prelli F. The defect in mu heavy chain disease protein GLI. J Immunol. (1976) 116:1194–5. 815437

[4] Hamers-Casterman C Atarhouch T Muyldermans S Robinson G Hamers C Songa EB. Naturally occurring antibodies devoid of light chains. Nature. (1993) 363:446–8. 10.1038/363446a0 8502296

[5] Muyldermans S. Single domain camel antibodies: current status. Rev Mol Biotechnol. (2001) 74:277–302. 10.1016/S1389-0352(01)00021-6 11526908

[6] Seligmann M Mihaesco E Preud'homme JL Danon F Brouet JC. Heavy chain diseases: current findings and concepts. Immunol Rev. (1979) 48:145–67. 10.1111/j.1600-065X.1979.tb00302.x 121099

[7] Sitia R Braakman I. Quality control in the endoplasmic reticulum protein factory. Nature. (2003) 426:891. 10.1038/nature02262 14685249

[8] Ellgaard L McCaul N Chatsisvili A Braakman I. Co- and post-translational protein folding in the ER. Traffic. (2016) 17:615–38. 10.1111/tra.12392 26947578

[9] Feige MJ Hendershot LM Buchner J. How antibodies fold. Trends Biochem Sci. (2010) 35:189–98. 10.1016/j.tibs.2009.11.005 20022755 PMC4716677

[10] Feige MJ Groscurth S Marcinowski M Shimizu Y Kessler H Hendershot LM. An unfolded CH1 domain controls the assembly and secretion of IgG antibodies. Mol Cell. (2009) 34:569–79. 10.1016/j.molcel.2009.04.028 19524537 PMC2908990

[11] Wahner-Roedler DL Kyle RA. Heavy chain diseases In: Edmonson K Lebowitz H, editors. Williams Hematology. 9th Edn. New York, NY: McGraw-Hill (2016). p. 1803–12.

[12] Kyttaris VC. Systemic lupus erythematosus: from genes to organ damage. Methods Mol Biol. (2010) 662:265–83. 10.1007/978-1-60761-800-3_13 20824476 PMC3153363

[13] Leach IH Jenkins JS Murray-Leslie CF Powell RJ. Mu-heavy chain and monoclonal IgG K paraproteinaemia in systemic lupus erythematosus Rheumatology. (1987) 26:460–2. 10.1093/rheumatology/26.6.460 3120844

[14] Wardemann H Yurasov S Schaefer A Young JW Meffre E Nussenzweig MC. Predominant autoantibody production by early human B cell precursors. Science. (2003) 301:1374–7. 10.1126/science.1086907 12920303

[15] Li S Yu Y Yue Y Liao H Xie W Thai J. Autoantibodies from single circulating plasmablasts react with citrullinated antigens and porphyromonas gingivalis in rheumatoid arthritis. Arthr Rheumatol. (2015) 68:614–26. 10.1002/art.39455 26474325 PMC5770231

[16] Liao H Yu Y Li S Yue Y Tao C Su K. Circulating plasmablasts from chronically human immunodeficiency virus-infected individuals predominantly produce polyreactive/autoreactive antibodies. Front Immunol. (2017) 8:1691. 10.3389/fimmu.2017.01691 29270169 PMC5723652

[17] Wardemann H Hammersen J Nussenzweig MC. Human autoantibody silencing by immunoglobulin light chains. J Exp Med. (2004) 200:191–9. 10.1084/jem.20040818 15263026 PMC2212019

[18] Clarke SC Ma B Trinklein ND Schellenberger U Osborn MJ Ouisse LH. Multispecific antibody development platform based on human heavy chain antibodies. Front Immunol. (2019) 9:3037. 10.3389/fimmu.2018.03037 30666250 PMC6330309

[19] Palade G. Intracellular aspects of the process of protein synthesis. Science. (1975) 189:347–58. 10.1126/science.1096303 1096303

[20] Lee JG Takahama S Zhang G Tomarev SI Ye Y. Unconventional secretion of misfolded proteins promotes adaptation to proteasome dysfunction in mammalian cells. Nat Cell Biol. (2016) 18:765–76. 10.1038/ncb3372 27295555 PMC10701763

[21] Chardin P McCormick F. Brefeldin A: the advantage of being uncompetitive. Cell. (1999) 97:153–5. 10.1016/S0092-8674(00)80724-2 10219235

[22] Mollenhauer HH James Morré D Rowe LD. Alteration of intracellular traffic by monensin; mechanism, specificity and relationship to toxicity. Biochim Biophys Acta. (1990) 1031:225–46. 10.1016/0304-4157(90)90008-Z 2160275 PMC7148783

[23] Behnke J Feige MJ Hendershot LM. BiP and its nucleotide exchange factors Grp170 and Sil1: mechanisms of action and biological functions. J Mol Biol. (2015) 427:1589–608. 10.1016/j.jmb.2015.02.011 25698114 PMC4356644

[24] Han J Back SH Hur J Lin YH Gildersleeve R Shan J. ER-stress-induced transcriptional regulation increases protein synthesis leading to cell death. Nat Cell Biol. (2013) 15:481–90. 10.1038/ncb2738 23624402 PMC3692270

[25] Cheng Z Teo G Krueger S Rock TM Koh HWL Choi H. Differential dynamics of the mammalian mRNA and protein expression response to misfolding stress. Mol Syst Biol. (2016) 12:855. 10.15252/msb.20156423 26792871 PMC4731011

[26] Pisetsky DS. Anti-DNA antibodies–quintessential biomarkers of SLE. Nat Rev Rheumatol. (2016) 12:102–10. 10.1038/nrrheum.2015.151 26581343

[27] Sonenshein GE Siekevitz M Siebert GR Gefter ML. Control of immunoglobulin secretion in the murine plasmacytoma line MOPC 315. J Exp Med. (1978) 148:301–12. 10.1084/jem.148.1.301 97359 PMC2184924

[28] Dul JL Aviel S Melnick J Argon Y. Ig light chains are secreted predominantly as monomers. J Immunol. (1996) 157:2969–75. 8816404

[29] Köhler G Howe SC Milstein C. Fusion between immunoglobulin-secreting and nonsecreting myeloma cell lines. Eur J Immunol. (1976) 6:292–5. 10.1002/eji.1830060411 825374

[30] Franklin EC Frangione B Prelli F. The defect in mu heavy chain disease protein GLI. J Immunol. (1976) 116:1194–5. 815437

[31] Alexander A Anicito I Buxbaum J. Gamma heavy chain disease in man. Genomic sequence reveals two noncontiguous deletions in a single gene. J Clin Investig. (1988) 82:1244–52. 10.1172/JCI113722 3139711 PMC442675

[32] Blanc MR Anouassi A Ahmed Abed M Tsikis G Canepa S Labas V. A one-step exclusion-binding procedure for the purification of functional heavy-chain and mammalian-type gamma-globulins from camelid sera. Biotechnol Appl Biochem. (2009) 54:207–12. 10.1042/BA20090208 19824883

[33] Iijima M Sekiguchi N Nagata A Wagatsuma M Midorikawa K Kurimoto M. Gamma heavy chain disease with T-cell large granular lymphocytic leukemia: a case report and review of the literature. Int Med. (2016) 55:399–403. 10.2169/internalmedicine.55.5042 26875967

[34] Muyldermans S. Nanobodies: natural single-domain antibodies. Ann Rev Biochem. (2013) 82:775–97. 10.1146/annurev-biochem-063011-092449 23495938

[35] Buxbaum JN. Abnormal immunoglobulin synthesis in monoclonal immunoglobulin light chain and light and heavy chain deposition disease. Amyloid J Protein Folding Dis. (2001) 8:84–93. 10.3109/13506120109007349 11409038

[36] Shusta EV Kieke MC Parke E Kranz DM Wittrup KD. Yeast polypeptide fusion surface display levels predict thermal stability and soluble secretion efficiency. J Mol Biol. (1999) 292:949–56. 10.1006/jmbi.1999.3130 10512694

