# Supplementary material for: Production of Autoreactive Heavy Chain-Only Antibodies in Systemic Lupus Erythematosus
Source: Front Immunol. 2020 May 5;11:632. doi: 10.3389/fimmu.2020.00632 (PMC7214812; doi:10.3389/fimmu.2020.00632)
Supplement: Supplementary file 1 [file Table_1.DOCX]

**Table S1 Demographics and clinical information of study subjects**

|  | *Subject ID* | | *Age* | *Gender* | | *Race* | *SLEDAI* | *ANA* |
| --- | --- | --- | --- | --- | --- | --- | --- | --- |
| *SLE* | | *S1* | *40* | | *F* | *African American* | *4* | + |
|  | | *S3* | *29* | | *M* | *Caucasian* | *4* | + |
|  | | *S4* | *36* | | *F* | *African American* | *6* | + |
|  | | *S8* | *41* | | *F* | *African American* | *0* | + |
|  | | *S12* | *28* | | *F* | *African American* | *2* | + |
|  | | *S21* | *24* | | *F* | *Hispanic* | *16* | + |
|  | | *S34* | *55* | | *F* | *African American* | *11* | + |
|  | | *S35* | *42* | | *F* | *African American* | *9* | + |
| *control* | | *U78* | *38* | | *F* | *Caucasian* | *-* | - |
|  | | *U95* | *29* | | *M* | *African American* | *-* | - |
|  | | *U110* | *50* | | *F* | *African American* | *-* | - |
|  | | *U111* | *51* | | *F* | *African American* | *-* | - |
