# Supplementary material for: Production of Autoreactive Heavy Chain-Only Antibodies in Systemic Lupus Erythematosus
Source: Front Immunol. 2020 May 5;11:632. doi: 10.3389/fimmu.2020.00632 (PMC7214812; doi:10.3389/fimmu.2020.00632)
Supplement: Supplementary file 2 [file Table_2.DOCX]

**Table S2 Number of antibodies obtained from each individual**

| *Control* | *Antibody* | *SLE* | *Antibody* |
| --- | --- | --- | --- |
| *U78* | *24* | *S1* | *27* |
| *U95* | *26* | *S3* | *29* |
| *U110* | *22* | *S4* | *35* |
| *U111* | *27* | *S8* | *32* |
| *Total* | *99* | *S12* | *27* |
|  |  | *S21* | *26* |
|  |  | *S34* | *20* |
|  |  | *S35* | *26* |
|  |  | *Total* | *222* |
