# Supplementary material for: Production of Autoreactive Heavy Chain-Only Antibodies in Systemic Lupus Erythematosus
Source: Front Immunol. 2020 May 5;11:632. doi: 10.3389/fimmu.2020.00632 (PMC7214812; doi:10.3389/fimmu.2020.00632)
Supplement: Supplementary file 3 [file Table_3.DOCX]

## Table S1. Demographics and clinical information of study subjects.

## Table S2. Number of antibodies obtained from each individual.

## Figure S1. SLE HCAbs are assembled and transported through the canonical ER-Golgi protein transport and secretion pathway.

(A) BFA blocks transport of SLE HCAbs from ER to Golgi. BFA is brefeldin A, a Golgi vesicle-mediated protein transport inhibitor from ER to Golgi.. HC1 is the SLE (B) Monensin blocks transport of SLE HCAbs from Golgi to extracellular environment. Monensin inhibits trans-Golgi transport by collapsing intracellular Na^+^ and H^+^ gradients necessary for protein transport. F9 and H6 are SLE HCAbs with high secretory ability.A1 is the conventional heavy chain with no secretory ability.

**Figure S2. V_H_, D_H_, and J_H_ gene usages, HCDR3 length influence in 222 SLE heavy chains.**

(A) V_H_, (B) D_H_, and (C) J_H_ gene usages between non-secreted heavy chain group and secreted heavy chain group. (D) Relationship between the length of CDR3 and secretion efficiency.
