# Supplementary figures and images for: Production of Autoreactive Heavy Chain-Only Antibodies in Systemic Lupus Erythematosus
Source: Front Immunol. 2020 May 5;11:632. doi: 10.3389/fimmu.2020.00632 (PMC7214812; doi:10.3389/fimmu.2020.00632)

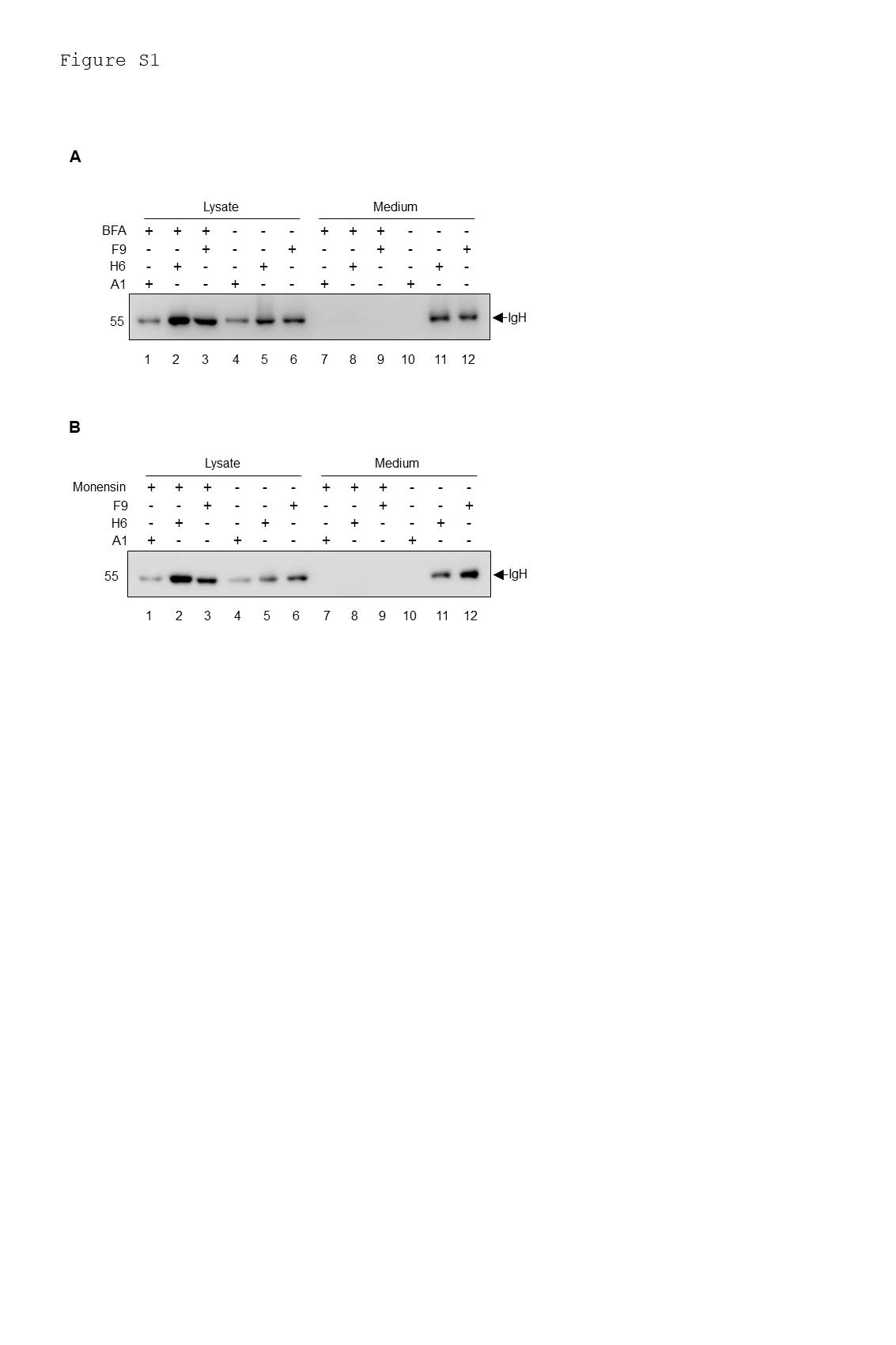

Supplement: Supplementary file 4 [file Image_1.JPEG]

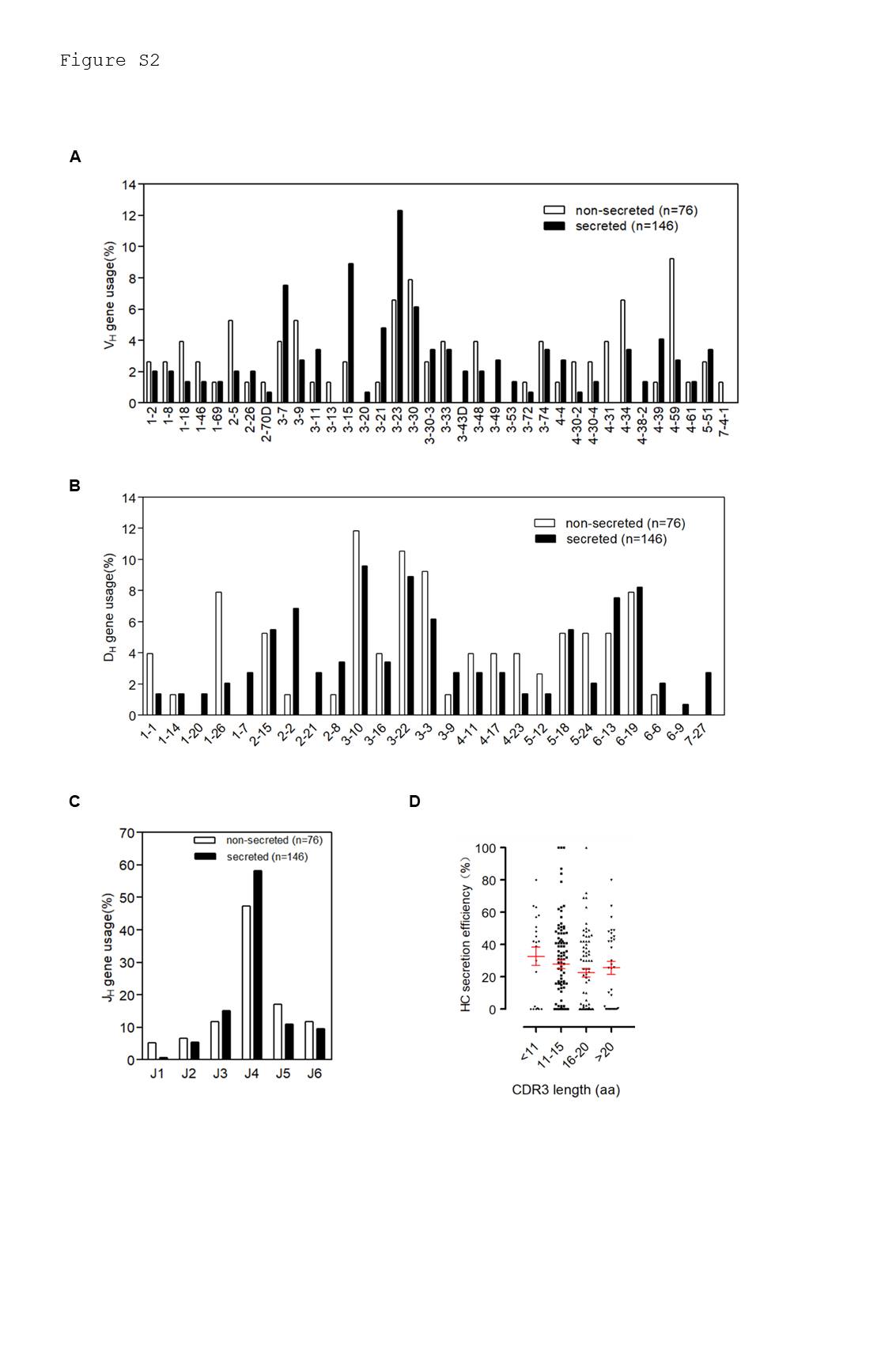

Supplement: Supplementary file 5 [file Image_2.JPEG]
